# Endothelial function as predictor in patients with coronary syndrome treated by percutaneous coronary intervention

**DOI:** 10.1042/BSR20180732

**Published:** 2018-10-15

**Authors:** Xiaofeng Cheng, Yun He, Huaping Fan, Ting Liu, Wenxu Pan, Ke Wang, Jun Jin

**Affiliations:** 1 Institute of Cardiovascular Diseases of PLA, Chongqing 400038, China; 2 Department of Cardiology, Xinqiao Hospital, Army Medical University (Third Military Medical University), Chongqing 400038, China

**Keywords:** acute coronary syndrome, endothelial function, major cardiovascular events, RH-PAT index

## Abstract

We aimed at identifying the predictive role of endothelial function assessed by the RH-PAT index (RHI) for future major cardiovascular events (MACEs) in acute coronary syndrome (ACS) patients treated with percutaneous coronary intervention (PCI). We measured RHI in 308 subjects with ACS, and they were divided into the normal endothelial function (NEF) group and the endothelial dysfunction (DEF) group according to the RHI. The subjects were followed up for a mean of 16 months (interquartile range [IQR]: 14–20 months) after PCI treatment, and their MACEs were also recorded. Cumulative incidence curves were constructed for time-to-event variables with Kaplan–Meier estimates and compared using the log-rank test. The overall incidence of MACEs was 25.39% in the DEF group and 15.96% in the NEF group (*P*<0.05). Kaplan–Meier analysis also demonstrated a significantly higher probability of MACEs in the DEF group than in the NEF group (log-rank test: *P*<0.05). Multivariate Cox hazard analysis identified RHI (Model 2, adjusted by blood pressure, hazard ratio [HR]: 0.425; 95% confidence interval [CI]: 0.198–0.914; *P*=0.029) and SYNTAX score (HR: 1.043; 95% CI: 1.019–1.067; *P*<0.001) as independent predictors of future MACEs after PCI treatment in ACS patients. Endothelial function measured by reactive hyperemia-peripheral arterial tonometry (RH-PAT) is impaired in ACS subjects treated with PCI. The RHI was an independent predictor of MACEs, suggesting that RHI may be useful as a candidate biomarker in the risk stratification of patients with ACS after PCI treatment.

## Introduction

Endothelial dysfunction (DEF), including coronary DEF and peripheral DEF, has been demonstrated to be an essential step in the initiation and progression of atherosclerosis [1,2]. It has also been considered to be a key process in atherogenesis and to contribute to the development of clinical cardiovascular diseases, including acute coronary syndrome (ACS) [3–6]. Although endothelial function studies in the past few decades have mainly focused on the coronary circulation, DEF in the peripheral arteries has also attracted considerable attention [7,8]. It has been widely indicated that DEF of peripheral arteries is strongly associated with coronary artery atherosclerosis and is an independent prognostic predictor of coronary artery disease (CAD), heart failure, and cerebrovascular diseases [2,7,9,10].

To date, various non-invasive assessments of peripheral artery endothelial function have been used extensively in clinic research and practice [2]. The high-resolution ultrasonographic measurement of brachial artery flow-mediated dilatation (FMD) was initially applied frequently to evaluate endothelial function and has been indicated to be correlated with coronary DEF and cardiovascular diseases. FMD measured in the forearm provides information that predicts the extent and severity of coronary atherosclerosis and is correlated with coronary endothelial function [11–14]. However, though this method provides information on the ‘recruitability’ of endothelial function, it does not take into account resting endothelial activity [15].

Subsequently, a novel non-invasive, automated, quantitative, and reproducible clinical examination for the evaluation of peripheral endothelial function, known as reactive hyperemia-peripheral arterial tonometry (RH-PAT), has been proposed [16–18]. It has identified invasively proven coronary DEF and ischemic heart disease while also predicting future cardiovascular events. Furthermore, the reactive hyperemia index (RHI) measured by RH-PAT serves as an excellent marker for cardiovascular events in patients in different stages of the cardiovascular disease continuum [19–22].

The impairment of reactive hyperemia (reduced RHI) in CAD patients has been observed previously [7,20]. Previous studies have also revealed associations between RHI and cardiovascular diseases, including the specific and emergent type, ACS, which requires early and emergent treatment. Despite advances in pharmacological therapy and percutaneous coronary intervention (PCI), recurrent major adverse cardiac events (MACEs) still occur in patients with ACS [23].

Although several studies have indicated the practical applications of RH-PAT, the role of RHI in MACEs among ACS patients treated with PCI has not been confirmed. In addition, the Framingham Heart Study revealed no statistically significant relationship between RHI and FMD, indicating that RHI and FMD represent distinct aspects of or risk factors for endothelial function and cardiovascular diseases [24]. RH-PAT may measure components of vasodilation that are not reflected in FMD, providing at least a theoretical basis for a more comprehensive assessment of vascular function [24]. Thus, we postulate that endothelial function measured by RH-PAT is impaired in ACS patients who are treated with PCI and may be a predictor of MACEs in this population.

## Methods

### Study design and population

**Figure 1:**
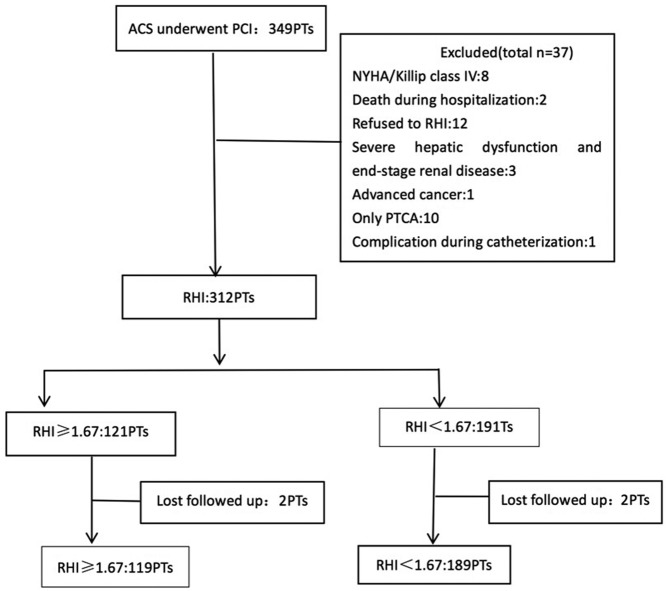
Study profile The flow chart of the present study showed our procedure in the performing this research.

The present study is a prospective, observational, single-center study of all consecutive patients with ACS treated with PCI at Xinqiao Hospital, a tertiary hospital located in the Shapingba district in Chongqing City, from 1 December 2015 to 30 September 2017. The inclusion criterion was ACS patients who were treated with PCI. The exclusion criteria were as follows: NYHA/Killip class IV (*n*=8), balloon angioplasty only without stent implantation (*n*=10), death during hospitalization (*n*=2), severe hepatic dysfunction and end-stage renal disease (*n*=3), advanced cancer (*n*=1), refused to RHI (*n*=12), and complication during catheterization (*n*=1), as shown in Figure 1. The study complied with the Declaration of Helsinki with respect to human investigations was approved by an institutional review committee, and was conducted in accordance with the guidelines of the ethics committee of Xinqiao Hospital. All the subjects provided written informed consent.

### Measurement of RHI

**Figure 2:**
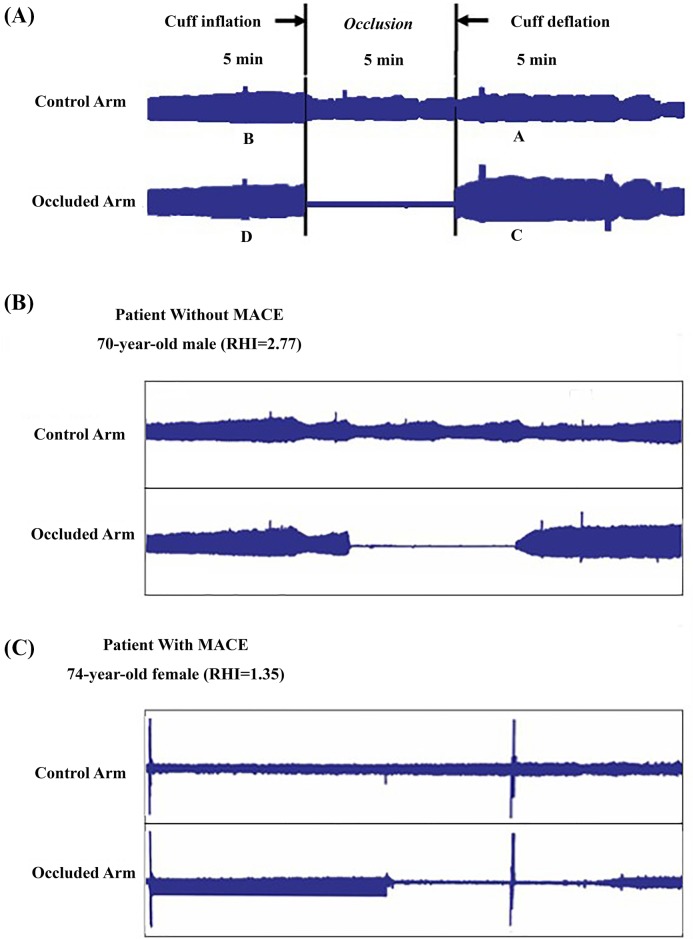
A representative for the measurement of RHI (**A**) RH-PAT ratio calculated: RH-PAT ratio = (C/D)/(A/B). Representative results of RH-PAT of subjects without cardiovascular events (**B**) and (**C**) those with cardiovascular events.

RHI was measured using the RH-PAT system (EndoPAT 2000; Itamar Medical, Caesarea, Israel) with the following procedures 48–72 h after PCI treatment. All measurements were performed in the early morning in a dedicated laboratory after fasting for at least 8 h. Medications containing long-lasting nitroglycerin, anti-ischemic, and anti-hypertensive medications for 24 h, and short-lasting nitroglycerin were withheld 1 h before the examination. The patients also had to refrain from caffeine consumption, smoking, and vasoactive medications. Before any measurements, the patients had an acclimatization period of 20 min in a quiet room, lying in a hospital bed at an ambient temperature of 21–24°C. The RH-PAT measurement protocol has previously been reported in detail [7]. Briefly, measurements were performed by the use of probes on the index fingers of both the study and control arms. A blood pressure cuff was placed on one upper arm with the contralateral arm serving as a control. The finger pulse wave amplitude was assessed with the EndoPAT-2000 sensing device and finger plethysmographic probes at baseline and during ischemia-induced hyperemia. Baseline measurements were recorded for 5 min prior to ischemia induction by inflating a blood pressure cuff on the upper arm of the study arm for 5 min to suprasystolic pressures. All subjects underwent RHI evaluation, and a representative figure is shown in Figure 2.

In the present study, RHI was calculated as the ratio of the mean hyperemic pulse wave analysis results over a period of 60 s, beginning at 90 s after cuff deflation, divided by the baseline pulse wave analysis results (mean baseline measurements for 2.5 min), and it was normalized to the concurrent measurements of the control arm. Participants were divided into two groups according to RHI: normal group (RHI ≥ 1.67, normal endothelial function [NEF] group) and abnormal RHI group (RHI < 1.67, DEF group) [22].

### Echocardiogram examination

Each subject received an echocardiogram examination (ultrasonography system, CX50, Philips, U.S.A.) in a quiet room in the supine position. The end-diastolic internal diameters of the left atrium (LA), left ventricle (LV), right atrium (RA), right ventricle (RV), and pulmonary artery (PA), in addition to the stroke volume (SV) and left ventricle ejection fraction (LVEF), were measured and recorded.

### Biomarker variables determination

Venous blood samples were obtained from subjects in the morning after an overnight fast (at least 12 h) within 24 h after admission. Plasma triglyceride (TG), low-density lipoprotein cholesterol (LDL-C), and high-density lipoprotein cholesterol (HDL-C) concentrations were assessed from venous blood samples using commercially available ELISA kits (Roche Diagnostics GmbH, Mannheim, Germany). The concentration of plasma creatinine (Cr) was measured by an enzymatic assay (Roche Diagnostics GmbH). All of the biochemical variables were measured from blood specimens in the Clinical Laboratory Department, Xinqiao Hospital.

### Coronary angiography

Coronary angiography was performed by a standard technique using 4–7 French right and left catheters through a femoral or radial artery approach. Clinically significant CAD was defined by the presence of a coronary lesion resulting in luminal stenosis ≥70% (≥50% at the left main coronary artery) or fractional flow reserve ≤0.80 in one or more major coronary arteries or their major branches. The PCI treatments were performed according to the guidelines on treatments of ST-segment elevation myocardial infarction (STEMI) [25] and NSTE-ACS recommended by Chinese Society of Cardiology [26].

### Clinical follow-up

After PCI treatment, the subjects were prospectively followed monthly at the outpatient clinic of Xinqiao Hospital until 30 September 2017 or until an endpoint event occurred. The MACE endpoints included cardiovascular death, acute myocardial infarction (AMI), target vessel revascularization (TVR), non-fatal ischemic stroke, and cardiac hospitalization. MACEs in subjects were evaluated and confirmed according to their medical records. Moreover, the first MACE for a subject was considered in the analysis if he/she suffered from more than two types of MACEs.

### Statistical analysis

Continuous variables with a normal distribution were expressed as the means ± S.D. and were compared using unpaired Student’s *t* test or one-way ANOVA, whereas the remaining variables with a non-normal distribution were compared using the Wilcoxon rank sum test. Dichotomous variables were assessed by the Chi-square test or Fisher’s exact test. Univariate and multivariate Cox regression analyses were performed to assess the effect of DEF on MACEs and to identify independent predictors of MACEs. Variables with *P* values less than 0.1 in the analyses, including differences between MACEs and non-MACEs groups and univariate Cox regression, were included into the multivariate Cox regression analysis. The variables with closely associations were analyzed in the regression independently (Model 1: the hypertension cases as a dichotomy variable selected into the regression; Model 2: included SBP and DBP into the analysis instead of hypertension). Hazard ratios (HRs) were calculated with 95% confidence intervals (95% CIs). Cumulative incidence curves were constructed for time-to-event variables with Kaplan–Meier estimates and compared using the log-rank test. The statistical analysis was performed with the statistical software ‘EZR’ (Easy R), which is based on R and R commander, and SPSS 20.0 for windows.

## Results

A total of 312 of the 349 ACS patients who were treated with PCI underwent the endothelial function assessment by EndoPAT. Finally, outpatient follow-up was completed in 308 patients, whereas four patients were lost to follow-up during the median follow-up period of 16 months (interquartile range [IQR]: 14–20 months).

Baseline clinical characteristics were compared between the DEF group (*n*=191) and NEF group (*n*=121, Table 1). There were no significant differences in age, gender, BMI, incidence of diabetes mellitus, or percentages of smoking and alcohol consumption between the two above-mentioned groups. The LVEF of the subjects was significantly lower in the DEF group than the NEF group (*P*<0.05). A calcium channel blocker (CCB) was used in 12.04% of the patients in the DEF group compared with 22.31% in the NEF group (*P*<0.05). Other medication use, including angiotensin-converting enzyme inhibitors/angiotensin receptor blockers (ACEIs/ARBs), β-blockers, antiplatelets, statins, and ezetimibe, showed no significant differences between the NEF and DEF groups (*P*>0.05). Moreover, the brain-type natriuretic peptide (BNP) level in the DEF group was significantly higher than that in the NEF group (*P*=0.035), while the systolic blood pressure was lower in the DEF group (*P*=0.013). In addition, the number of patients with STEMI was also higher in the DEF group than in the NEF group (*P*=0.003).

**Table 1:** Baseline characteristics of the subjects

Variables	NEF group (*n*=121)	DEF group (*n*=191)	*P* value	Power of test
Age, mean (SD), years	59.44 ± 9.55	60.63 ± 9.94	0.291	0.183
Male gender, *n* (%)	89 (73.55%)	151 (79.06%)	0.273	
BMI, mean (SD), kg/m^2^	24.90 ± 3.05	24.32 ± 2.82	0.084	0.389
LV mean (SD), mm	46.47 ± 5.02	47.64 ± 5.98	0.135	0.458
LVEF, mean (SD), %	62.66 ± 6.00	59.67 ± 9.39	0.002^†^	0.928
BNP, Median (IQR), pg/ml	37.40 (139.20)	68.30 (188.00)	0.035*	
Peripheral vascular disease, *n* (%)	77 (63.63%)	120 (62.83%)	0.905	
Pre-PCI, *n* (%)	18 (14.88%)	15 (7.85%)	0.059	
Pre-CABG, *n* (%)	1 (0.83%)	0 (0.00%)	0.388	
Current smoking, *n* (%)	56 (46.28%)	107 (56.02%)	0.104	
Creatinine mean (SD), μmol/l	75.45 ± 17.33	77.36 ± 19.06	0.386	0.149
ALT, mean (SD), IU/l	30.60 ± 20.30	35.48 ± 28.11	0.095	0.425
AST, mean (SD), IU/l	25.89 ± 14.62	28.86 ± 21.45	0.177	0.305
Hypertension, *n* (%)	76 (62.81%)	110 (57.59%)	0.408	
Systolic BP, mean (SD), mm Hg	133.35 ± 18.96	127.98 ± 18.66	0.013*	0.686
Diastolic BP, mean (SD), mm Hg	79.45 ± 11.23	77.05 ± 12.03	0.075	0.430
TC, mean (SD), mmol/l	4.02 ± 1.11	3.98 ± 1.14	0.774	0.061
TG, mean (SD), mmol/l	1.55 ± 0.89	1.67 ± 1.01	0.263	0.195
LDL-C, mean (SD), mmol/l	2.61 ± 0.93	2.60 ± 0.83	0.760	0.051
HDL-C, mean (SD), mmol/l	1.00 ± 0.21	1.01 ± 0.25	0.973	0.067
Diabetes mellitus, *n* (%)	30 (24.79%)	60 (31.41%)	0.202	
HbA1c, mean (SD), %	6.57 ± 1.55	6.74 ± 1.51	0.433	0.158
Diagnosis			0.014*	
STEMI, *n* (%)	15 (12.39%)	50 (26.18%)	0.003*	
NSTEMI, *n* (%)	8 (6.61%)	12 (6.28%)	0.136	
UA, *n* (%)	98 (81.00%)	129 (67.54%)	0.784	
Medication				
ACEI/ARB, *n* (%)	88 (72.72%)	127 (66.49%)	0.261	
CCB, *n* (%)	27 (22.31%)	23 (12.04%)	0.018	
β-Blockers, *n* (%)	92 (76.03%)	147 (76.96%)	0.891	
Antiplatelet				
Aspirin + Clopidogrel, *n* (%)	45 (37.19%)	68 (35.60%)	0.810	
Aspirin + Ticagrelor, *n* (%)	76 (62.81%)	123 (64.40%)	0.810	
Trimetazidine, n (%)	70 (57.85%)	102 (53.40%)	0.559	
Statins, *n* (%)	121 (100.00%)	191 (100.00%)	1.000	

Abbreviations: ALT, aspartate aminotransferase; AST, aspartate transaminase; BMI, body mass index; CABG, cardiac artery bypass graft; GRACE, Score, Global Registry of Acute Coronary Events Risk Score; LV, left ventricular end diastolic dimension; NSTEMI, non ST-elevation myocardial infarction; PCI, percutaneous coronary interventional treatment; TC, total cholesterol; UA, unstable angina pectoris. *P≤0.05; ^†^*P*≤0.01.

Table 2 showed the differences in the coronary artery lesion characteristics of subjects between the DEF and NEF groups. There were no significant differences in SYNTAX score, the percent of thrombus lesions, number of diseased coronary vessels, and other parameters between the NEF and DEF groups (all *P* values > 0.05).

**Table 2:** Patient coronary artery lesion characteristics

	NEF group (*n*=121)	DEF group (*n*=191)	*P* value	Power for test
Number of diseased coronary vessels			0.052	
One, *n* (%)	51 (42.15%)	55 (28.80%)		
Two, *n* (%)	30 (24.79%)	57 (29.84%)		
Three, *n* (%)	40 (33.06%)	79 (41.36%)		
SYNTAX score (SD)	16.21 ± 10.20	18.30 ± 9.64	0.069	0.434
Thrombus lesions, *n* (%)	8 (6.61%)	22 (11.52%)	0.172	
Chronic total occlusion, *n* (%)	16 (13.22%)	32 (16.75%)	0.426	
Curve lesions, *n* (%)	4 (3.30%)	10 (5.24%)	0.577	
Calcificaton lesions, *n* (%)	14 (11.57%)	33 (17.28%)	0.129	
LM lesions, *n* (%)	20 (16.52%)	25 (13.09%)	0.412	
Number of stents, *n*	1.83 ± 0.97	1.93 ± 0.98	0.391	
Medina, 1,1,1, *n* (%)	32 (26.45%)	55 (28.80%)	0.699	
T/TAP technique, *n* (%)	3 (2.48%)	2 (1.05%)	0.380	
Crush/mini crush technique, *n* (%)	2 (1.65%)	6 (3.14%)	0.491	
BT/ BSKT technique, *n* (%)	11 (9.90%)	9 (4.71%)	0.155	

Abbreviations: BSKT, balloon stent kissing technique; JBT, jailed-balloon technique; LM, left main coronary artery; SYNTAX score, synergy between PCI with taxus and cardiac surgery score; T/TAP, T stenting and small protrusion technique.

**Figure 3:**
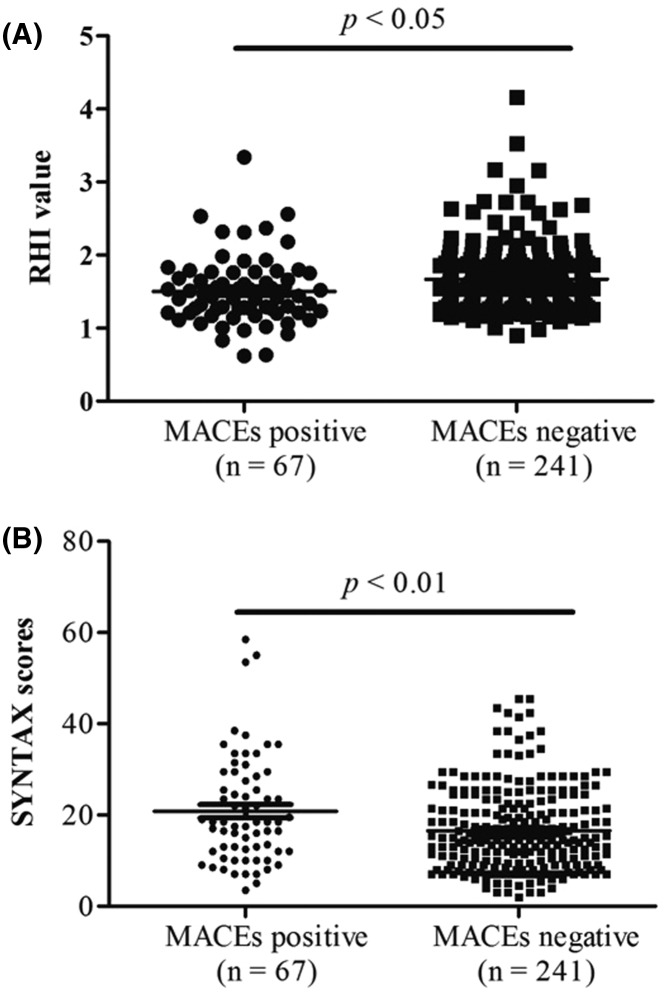
Differences of RHI and SYNTAX score between MACEs and non-MACEs groups RHI (**A**) value was lower (**B**) was significantly higher in MACEs subjects than non-MACE ones.

Patients were followed up for a median period of 16 months, during which 67 (21.75%) patients suffered from a MACE. The incidence of cardiovascular events in the DEF group (*n*=48, 25.39%) was significantly higher than that in the NEF group (*n*=19, 15.96%, *P*<0.05), as shown in Table 3. Specifically, non-fatal ischemic stroke occurred in seven patients (3.70%) in the DEF group, while no patients suffered from stroke in the NEF group (*P*<0.05). However, the incidences of other types of MACEs, including AMI, TVR, cardiac death, and cardiac hospitalization, showed no difference between the DEF group and NEF group (*P*>0.05). Additionally, the RHI in patients with MACEs was obviously lower than that in patients without MACEs (*P*<0.05, Figure 3A).

**Table 3:** Estimated incidence of major adverse cardiac events in patients undergoing PCIs

	Overall (*n*=308)	NEF group (*n*=119)	DEF group (*n*=189)	HR (95% CIs)	*P* value
AMI, *n* (%)	13 (4.22%)	4 (5.04%)	9 (4.76%)	1.015 (0.969–1.063)	0.389
TVR, *n* (%)	4 (1.30%)	1 (0.84%)	3 (1.59%)	1.016 (0.998–1.034)	0.285
Cardiac death, *n* (%)	8 (2.60%)	1 (0.84%)	7 (3.70%)	1.029 (0.997–1.063)	0.157
Non-fatal ischemic stroke, *n* (%)	7 (2.27%)	0 (0.00%)	7 (3.70%)	∞ (1.010 -∞)	0.046*
Cardiac hospitalization, *n* (%)	35 (11.36%)	13 (10.92%)	22 (11.64%)	1.008 (0.929–1.094)	0.501
MACEs, *n* (%)	67 (21.75%)	19 (15.96%)	48 (25.39%)	1.142 (1.019–1.280)	0.036*

*P≤0.05.

The overall SYNTAX score was higher in subjects suffering from MACEs than in the non-MACEs subjects (*P*<0.05, Figure 3B). Furthermore, the LVEF and systolic BP were also significantly lower in the MACEs group than that in the non-MACEs group, while the diastolic BP was higher in the MACEs group (*P*<0.05, Supplementary Table S1). However, the coronary artery lesion characteristics showed no significant differences between the MACEs positive and MACEs negative groups (all *P* values <0.05, Supplementary Table S2).

Cox proportional hazards analyses were also performed to identify the associations of endothelial function, cardiovascular risk factors, and heart function with future MACEs after PCI treatment. Unadjusted Cox proportional hazards regression models showed that RHI (HR: 0.335, 95% CI: 0.167–0.675; *P*<0.05), LVEF (HR: 0.973, 95% CI : 0.948–0.998; *P*<0.05), and SYNTAX score (HR: 1.052, 95% CI: 1.029–1.075; *P*<0.01) were predictive of MACEs after PCI treatment (Table 4). However, multivariate regression (stepwise backward algorithm multivariate Cox proportional hazard analysis) revealed that RHI (Model 2 adjusted by blood pressure, HR: 0.425, 95% CI: 0.198–0.914; *P*=0.029) was an independent predictor of future MACEs after PCI treatment (Table 5). Furthermore, we have also identified that the SYNTAX score is also an independent predictor for MACEs (HR: 1.043; 95% CI: 1.019–1.067; *P*<0.001, Table 5).

**Table 4:** Cox proportional comparison hazards for future MACEs in patients after PCI

Variables	Univariate regression		
	HR	95% CI	*P* value
RHI	0.335	0.167–0.675	0.002^†^
Age (years old)	1.002	0.978–1.026	0.900
Male (yes)	0.745	0.431–1.288	0.292
Current smoker (yes)	0.859	0.529–1.395	0.539
Diabetes mellitus (yes)	1.397	0.836–2.337	0.202
Hypertension (yes)	1.661	0.982–2.811	0.059
Systolic BP (mm Hg)	1.002	0.989–1.015	0.798
Diastolic BP (mm Hg)	0.990	0.968–1.013	0.383
Heart rate (per beats/min)	0.997	0.977–1.017	0.753
LVEF (%)	0.973	0.948–0.998	0.036*
TG (mmol/l)	1.106	0.884–1.385	0.378
HDL (mmol/l)	1.838	0.696–4.851	0.219
LDL (mmol/l)	1.147	0.931–1.607	0.147
SYNTAX score	1.052	1.029–1.075	<0.001^†^
TC (mmol/l)	1.128	0.915–1.390	0.259
BNP (pg/ml)	1.001	1.000–1.001	0.098
Cr (μmol/l)	0.998	0.985–1.012	0.811
ALT (IU/l)	0.998	0.988–1.008	0.639
AST (IU/l)	0.999	0.988–1.009	0.814
Pre-PCI	0.473	0.171–1.304	0.148
Peripheral vascular disease	1.139	0.691–1.878	0.609

*P≤0.05; ^†^*P*≤0.01.

**Table 5:** Cox proportional comparison hazards for future MACEs in patients after PCI (multivariate regression)

Variables	Multivariate regression Model 1			Multivariate regression Model 2		
	HR	95% CI	*P*	HR	95% CI	*P*
RHI	0.415	0.195–0.884	0.023*	0.425	0.198–0.914	0.029*
Hypertension (yes)	1.153	0.686–1.939	0.591	–	–	–
Systolic BP (mm Hg)	–	–	–	1.011	0.994–1.029	0.202
Diastolic BP (mm Hg)	–	–	–	1.010	0.982–1.010	0.210
LVEF (%)	1.000	0.998–1.001	0.153	1.004	0.952–1.004	0.095
SYNTAX score	1.043	1.019–1.067	<0.001^†^	1.043	1.019–1.067	<0.001^†^
BNP (pg/ml)	1.000	0.998–1.001	0.593	1.000	0.998–1.001	0.527
BMI (kg/m^2^)	1.042	0.958–1.134	0.335	1.043	0.958–1.135	0.333
Number of diseased coronary vessels	0.897	0.673–1.197	0.461	0.897	0.675–1.196	0.465
Pre-PCI history	1.103	0.537–2.266	0.790	1.057	0.510–2.191	0.881
Creatinine, (μmol/l)	0.997	0.983–1.010	0.638	0.998	0.984–1.011	0.722

Model 1: The hypertension cases as a dichotomy variable selected into the regression; Model 2: Included SBP and DBP into the analysis instead of hypertension. *P≤0.05; ^†^*P*≤0.01.

**Figure 4:**
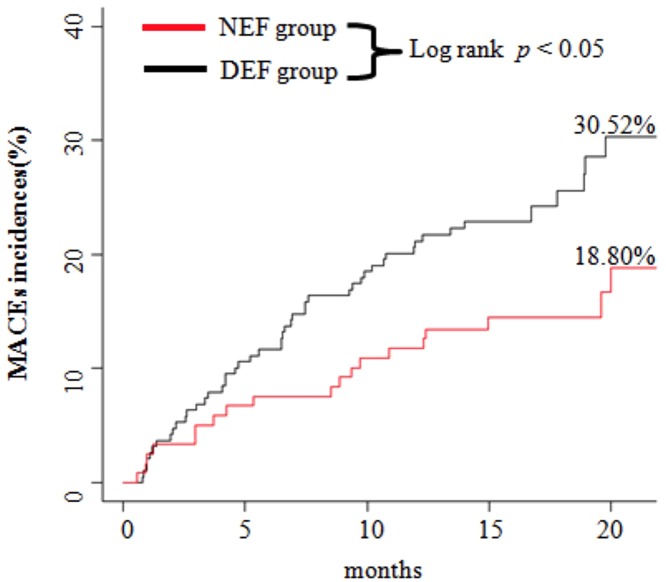
Kaplan–Meier curves of cumulative incidences of MACEs Subjects in the RHI < 1.67 group (DEF group) were shown to have a higher risk of MACEs after PCI treatment (*P*=0.008).

Additionally, Kaplan–Meier survival curves illustrated the relationship between RHI and MACEs (Figure 4). Compared with the RHI ≥ 1.67 group (NEF group) as a reference, subjects in the RHI < 1.67 group (DEF group) were shown to have a higher risk of MACEs after PCI treatment (*P*=0.008).

## Discussion

In the present study, we focused on the association between peripheral DEF and MACEs in a specific type of CAD patients; namely, patients with ACS after they received PCI treatment, in whom this association has not been previously reported. We found that the incidence of MACEs was significantly higher in the DEF group (RHI < 1.67) than in the NEF group among ACS patients who were treated with PCI during the follow-up period (median: 16 months). Furthermore, we also observed that the patients in the DEF group were characterized by a lower LVEF. Finally, we demonstrated that in addition to the traditional predictors, RHI was an independent predictor of future MACEs after PCI treatment in ACS patients.

As mentioned above, peripheral DEF plays a pivotal role in the development of clinical cardiovascular diseases [1,4,7]. Recently, a novel peripheral endothelial function assessment technique, RH-PAT, has been widely applied in clinical research and practice. Although both FMD and RHI were associated with CAD and were also predictors of cardiac events, the relationship between FMD and RHI has been controversial [2,27]. RH-PAT also has clear advantages over the established methods of coronary angiography and ultrasound used to measure endothelial function [2]. Thus, it is important to identify the roles of RHI in cardiovascular diseases. The associations between RHI and coronary blood flow, coronary DEF, coronary microvascular function, heart failure, and their future events have been reported. The results above were consistent with those of previous reports, in which RHI was shown to be a risk factor in addition to the traditional risk factors [22,28], which may provide guidance for ACS risk stratification.

In addition, we found that LVEF was much lower in the DEF group than in the NEF group, suggesting that cardiac function was also affected to some degree. The lower LVEF in the DEF group may be also a reason for the lower systolic BP in this group. Also of note is that BNP levels were significantly higher in the DEF group than in the NEF group, which supports the previously reported association between cardiac function and peripheral DEF [9]. This association may also be a mechanism underlying the predictive roles of RHI in future cardiovascular disease events. However, in the adjusted model, LVEF could not predict future MACEs after PCI treatment following RHI adjustment.

Interestingly, we found that in the coronary artery lesion characteristic analysis, there were no significant differences in the number of diseased coronary vessels, lesion types in coronary arteries (including the left main artery), number of implanted stents, calcification, occlusion cases, or other characteristics. Although ACS patients had the same baseline and major coronary artery lesion characteristics at the time of PCI treatment, patients in the DEF group had a poor prognosis and a higher incidence of MACEs in the future, implying that DEF may be a prognostic marker in ACS patients. This predictive role may be attributed to the higher incidence of STEMI in populations with peripheral DEF. Further analyses revealed that in the MACE subtype analysis, non-fatal ischemic stroke had a higher incidence in the DEF group, indicating DEF in the peripheral arteries as well as cerebral arterioles. In addition, in the populations with MACEs, RHI was significantly lower than that in the non-MACEs subjects, indicating associations between DEF and MACEs. The higher incidence of non-fatal ischemic stroke in the DEF group suggested that DEF may be a risk factor for cardiovascular diseases as well as cerebral artery atherosclerosis, microvascular diseases and thromboembolism or, consequently, stroke. This may provide insight into predicting or assessing cerebral artery atherosclerosis or DEF, which needs further methodological and basic mechanisms studies. Furthermore, the RHI assessment may also be part of the risk management, stratification of ischemic stroke and prediction of stroke.

Finally, we performed Cox regression analyses to identify predictors of MACEs in ACS patients after PCI treatment. In the univariate regression, RHI, SYNTAX score, and lower LVEF were predictors of MACEs. However, there were many differences in clinical characteristics, blood pressure, and medication between patients with normal and abnormal endothelial function, thus it is also possible that these factors influenced the endothelial function in the MACEs occurrence. Furthermore, in the adjusted regression, other variables were adjusted by the RHI and SYNTAX score, meaning that RHI and SYNTAX score were still independent predictors of MACEs during the 16-month (median) follow-up period in the PCI-treated ACS patients. Combined with the Kaplan–Meier survival analysis findings (patients in the DEF group had a higher risk of MACEs), RHI impairment could provide a role in peripheral DEF as an independent predictor of prognosis (MACEs) in PCI-treated ACS patients.

## Limitations

First, this was a single-center study with a limited sample size and number of events. Further large, multicenter studies with various ethnic groups are required.

Second, patients with hemodynamic instability or who died as a consequence of ACS before the RHI was measured were not included in the present study, which may lead to a reduced ability of the SYNTAX score to predict MACEs by excluding patients with serious CAD.

Third, our study excluded ACS patients with an unstable condition despite maximal therapy (NYHA class IV) due to difficulty in performing RHI measurements.

Fourth, we knew the RHI of patients during to follow for MACE, which makes the trial non-blind. Moreover, though this is a 5-year study, the current result can indicate that RHI can predict the MACE in these patients. Further prospective clinical trials are needed, as an observational study cannot determine causality between RHI and MACEs in the selected populations.

## Conclusion

Endothelial function measured by RH-PAT is impaired in ACS subjects treated with PCI. The founding that RHI was an independent predictor of MACEs, suggesting that RHI may be useful as a candidate biomarker in the risk stratification of patients with ACS after PCI treatment.

## Supporting information

**Table S1:** Baseline Characteristics of patients between MACEs and non-MACEs groups

**Table S2:** The coronary artery lesion characteristics of patients divided by MACEs

## Abbreviations

ACEI: angiotensin-converting enzyme inhibitor
ACS: acute coronary syndrome
AMI: acute myocardial infarction
BNP: brain-type natriuretic peptide
BP: Blood pressure
CAD: coronary artery disease
CCB: calcium channel blocker
CI: confidence interval
Cr: creatinine
DEF: endothelial dysfunction
FMD: flow-mediated dilatation
HDL-C: high-density lipoprotein cholesterol
HR: hazard ratio
IQR: interquartile range
LV: left ventricle
LVEF: left ventricle ejection fraction
LDL-C: low-density lipoprotein cholesterol
MACE: major cardiovascular event
NEF: normal endothelial function
NSTE-ACS: non ST-elevation acute coronary syndrome
NSTEMI: non ST-elevation myocardial infarction
PCI: percutaneous coronary intervention
RHI: reactive hyperemia index
RH-PAT: reactive hyperemia-peripheral arterial tonometry
STEMI: ST-segment elevation myocardial infarction
SYNTAX: Synergy Between PCI with Taxus and Cardiac Surgery
TG: triglyceride
TVR: target vessel revascularization
