# Supplementary material for: Endothelial function as predictor in patients with coronary syndrome treated by percutaneous coronary intervention
Source: Biosci Rep. 2018 Oct 15;38(5):BSR20180732. doi: 10.1042/BSR20180732 (PMC6435452; doi:10.1042/BSR20180732)
Supplement: Supplementary file 1 [file bsr20180732_Supp1.pdf]

Table S1. Baseline Characteristics of patients between MACEs and non-MACEs groups

Variable	MACEs positive （n = 67）	MACEs negative （n = 241）	p-value
Age, mean (SD), y	60.27±9.43	60.23±9.95	0.947
Male gender, n (%)	49(73.13%)	187(77.59%)	0.640
BMI, mean (SD), kg/m^2	24.09±3.18	24.70±2.84	0.084
LV mean (SD), mm	47.46±6.47	47.24±5.47	0.919
LVEF, mean (SD), %	58.94±10.01	61.41±7.84	0.001**
BNP, Median (IQR), pg/mL	62.70±183.58	57.70±144.73	0.360
Peripheral vascular disease	39(58.21%)	156(64.73%)	0.390
Pre-PCI, n (%)	4(5.97%)	36(14.94%)	0.064
Pre-CABG, n (%)	1(1.49%)	0(0.00%)	0.218
Current smoking, n (%)	33(49.25%)	129(53.53%)	0.518
Creatinine mean (SD), μmol/L	76.45±17.09	76.52±16.35	0.073
ALT, mean (SD), IU/L	33.10±27.85	33.11±23.98	0.516
AST, mean (SD), IU/L	29.12±20.87	27.20±18.46	0.516
Hypertension, n (%)	32(47.76%)	101(41.91%)	0.094
Systolic BP, mean (SD), mm Hg	131.30±21.67	130.11±18.28	0.008**
Diastolic BP, mean (SD), mm Hg	77.36±11.90	78.21±11.81	0.028*
TC, mean (SD), mmol/L	4.15±1.41	3.96±1.05	0.397
TG, mean (SD), mmol/L	1.75±1.12	1.62±0.94	0.819
LDL, mean(SD), mmol/L	2.68±0.96	2.59±0.69	0.608
HDL, mean(SD), mmol/L	1.02±0.24	1.00±0.24	0.432
Diabetes mellitus, n (%)	22(32.84%)	68(28.22%)	0.453
HbA1c, mean (SD), %	6.44±1.20	6.77±1.61	0.367
Diagnosis			0.981
STEMI, n (%)	14(20.90%)	50(20.75%)	
NSTEMI, n (%)	4(5.97%)	16(6.64%)	
UA, n (%)	49(73.13%)	175(72.61%)	
Medication			
ACEI/ARB, n (%)	44(65.67%)	168(69.71%)	0.553
CCB, n (%)	11(16.42%)	39(16.18%)	0.963
β-blockers, n(%)	55(82.09%)	180(74.69%)	0.256
Antiplatelet			

aspirin + Clopidogrel, n (%)	21(31.34%)	89(36.93%)	0.472
aspirin + Ticagrelor, n (%)	46(68.66%)	152(63.70%)	0.472
Trimetazidine, n (%)	38(56.72%)	133(55.19%)	0.890
Statins, n (%)	67(100%)	241(100%)	1.000

BMI, body mass index; LV, left ventricular end diastolic dimension; LVEF, left ventricular ejection fraction; PCIs, percutaneous coronary interventional treatments; CABG, cardiac artery bypass graft; ALT, aspartate aminotransferase; AST, aspartate transaminase; TC, total cholesterol; TG, triglyceride; HDL-C, high density lipoprotein cholesterol; LDL-C, low density lipoprotein cholesterol; STEMI, ST-Elevation Myocardial Infarction; NSTEMI, non ST-Elevation Myocardial Infarction; UA, unstable angina pectoris; CCB, calcium-channel blocker.

* $p \leq 0.05$; ** $p \leq 0.01$.

Table S2. The coronary artery lesion characteristics of patients divided by MACEs

	MACEs positive（n = 67）	MACEs negative（n = 241）	p value
Number of diseased coronary vessels			0.097
One, n (%)	17(25.37%)	87(36.10%)	
Two, n (%)	17(25.37%)	69(28.63%)	
Three, n (%)	33(49.26%)	85(35.27%)	
Thrombus lesions, n (%)	10(14.93%)	20(8.30%)	0.109
Chronic total occlusion, n (%)	12(17.91%)	36(14.94%)	0.570
Curve lesions, n (%)	3(4.48%)	11(4.56%)	1.000
Calcificaton lesions, n (%)	11(16.41%)	36(14.94%)	0.848
LM lesions，n (%)	13(19.40%)	32(13.28%)	0.240
Number of stents，n	1.83±0.97	1.93±0.98	0.391
Medina, 1,1,1，n (%)	17(25.37%)	69(28.63%)	0.647
T/TAP technique, n (%)	2(2.99%)	3(1.24%)	0.299
Crush/mini crush technique, n (%)	2(1.65%)	6(3.14%)	0.491
BT/ BSKT technique, n (%)	2(2.99%)	17(7.05%)	0.387

SYNTAX score: Synergy Between PCI With Taxus and Cardiac Surgery score; T/TAP:T stenting and small protrusion technique; JBT: jailed-balloon technique; BSKT: balloon stent kissing technique. LM：Left main coronary artery;
